# The p75 neurotrophin receptor localization in blood-CSF barrier: expression in choroid plexus epithelium

**DOI:** 10.1186/1471-2202-12-39

**Published:** 2011-05-11

**Authors:** Carlos Spuch, Eva Carro

**Affiliations:** 1 Neuroscience Group, Research Institute Hospital 12 de Octubre, Madrid, Spain; 2 Department of Pathology and Neuropathology, University Hospital of Vigo (CHUVI), Vigo, Spain; 3 Biomedical Research Networking Center in Neurodegenerative Diseases (CIBERNED), Madrid, Spain

## Abstract

**Background:**

The presence of neurotrophins and their receptors Trk family has been reported in the choroid plexus. High levels of Nerve Growth Factor (NGF), Neurotrophin-4 (NT-4) and TrkB receptor were detected, while nothing was know about p75 neurotrophin receptor (p75NTR) in the choroid plexus epithelial cells. In neurons, p75NTR receptor has a dual function: promoting survival together with TrkA in response to NGF, and inducing apoptotic signaling through p75NTR. We postulated that p75NTR may also affect the survival pathways in the choroid plexus and also undergoes regulated proteolysis with metalloproteases.

**Results:**

Here, we demonstrated the presence of p75NTR receptor in the choroid plexus epithelial cells. The p75NTR receptor would be involved in cell death mechanisms and in the damaged induced by amyloid beta (Aβ) in the choroid plexus and finally, we propose an essential role of p75NTR in the Aβ transcytosis through out choroid plexus barrier.

**Conclusions:**

The presence analysis reveals the new localization of p75NTR in the choroid plexus and, the distribution mainly in the cytoplasm and cerebrospinal fluid (CSF) side of the epithelial cells. We propose that p75NTR receptor plays a role in the survival pathways and Aβ-induced cell death. These data suggest that p75NTR dysfunction play an important role in the pathogenesis of brain diseases. The importance and novelty of this expression expands a new role of p75NTR.

## Background

The choroid plexus, located in the cerebral ventricles, is a highly vascularised tissue, in which blood microvessels are enclosed by a single layer of cubical epithelial cells [1]. Choroid plexus epithelial cells are closely connected to each other by tight junctions and constitute the structural basis of the blood-CSF barrier [2]. The barrier function can be subjected to modulation and thereby regulates the entry of physiologically important substances. However, choroid plexus epithelial cells are also an important target organ for polypeptides, with capacity to produce and secrete numerous biologically active neurotrophic factors into the CSF. In mammalian, brain CSF is produced by the choroid plexus, which not only regulates homeostasis in central nervous system (CNS), but also participates in neurohumoral brain modulation as well as in neuroimmune interaction. Several peptides are shown to be actively transported by the choroidal epithelial cells to the CSF and most of the transported hormones evidently have, at least, a hypothalamic destination. These peptides circulate throughout the brain and spinal cord, maintaining neuronal networks and associated cells [3].

Studies in the past few years have promoted insight into the molecular structure and function of the choroid plexus. Even modest changes in the choroid plexus can have far reaching effects and changes in the anatomy and physiology of the choroid plexus, and have been linked to several neurological disorders such as Alzheimer's disease or multiple sclerosis. Given a widely postulated role in neuronal cell survival, the p75NTR is also though to be associated with many neurodegenerative diseases.

The choroid plexus is involved in a variety of neurological disorders, including neurodegenerative, inflammatory, infectious, traumatic, neoplastic, and systemic diseases. It is well known that Aβ is accumulated in the choroid plexus of Alzheimer's disease patients [4]; there is also evidence that circulating Insulin-Like Growth Factor-I (IGF-I) participates in brain Aβ clearance by modulating choroid plexus function [5]. It has been published that substances, such as transthyrretin (TTR) or apolipoprotein J (ApoJ), are produced by the choroid plexus and secreted into the CSF [6]. In multiple sclerosis and in animal models of multiple sclerosis, the choroid plexus is the main route of leukocyte entry into the brain [7].

Many studies have demonstrated the presence of proteins and mRNA for a large number of cytokines, growth factors and hormones in the choroid plexus, for example: interleukin-1β [8], interleukin-6 [9], Tumor Necrosis Factor (TNF)-α [10], IGF-I [11], NGF [12], IGF-II [13], Transforming Growth Factor (TGF)-α [14], TGF-β [15], Vascular Endothelial Growth Factor (VEGF) [16], transferrin [17], TTR [6,18], gelsolin [19] and vasopressin [20]. Most of these substances have their own receptors in the choroid plexus [21]. The production and secretion of all these substances and their receptors are strongly associated with the health of central nervous system (CNS).

The mammalian neurotrophins comprise a family of related secreted factors required for differentiation, survival, development, and death of specific populations of neurons and non-neuronal cells. The effects of the neurotrophins (NGF, Brain-Derived Neurotrophin Factor (BDNF), NT-3, NT-4) are mediated by binding to TrkA, TrkB and TrkC receptor tyrosine kinases and to the p75NTR [22,23]. The Trk receptors play critical roles in mediating neuronal survival, growth and synaptic function [24]. The p75NTR serves as a receptor for the four mentioned neurotrophins and it is an important component of distinct cell surface signaling platforms, which induce apoptosis and neuronal growth inhibition [25].

The presence of the neurotrophins and their receptors has been investigated in the choroid plexus of rats and humans. The choroid plexus contains high levels of NGF, NT-4, and TrkB, and low levels of NT-3 and BDNF, while TrkA and TrkC levels remain undetectable [12].

Regarding the neurotrophins receptors in the choroid plexus, the expression of p75NTR has not been fully investigated. The present research shows the distribution of p75NTR in the epithelial cells of the choroid plexus and its possible role in the normal transportation of molecules of the blood-CSF-barrier. The importance and novelty of this expression expands a new role of p75NTR.

## Results

### 1. The p75 neurotrophin receptor is expressed in the epithelial cells from choroid plexus

**Figure 1:**
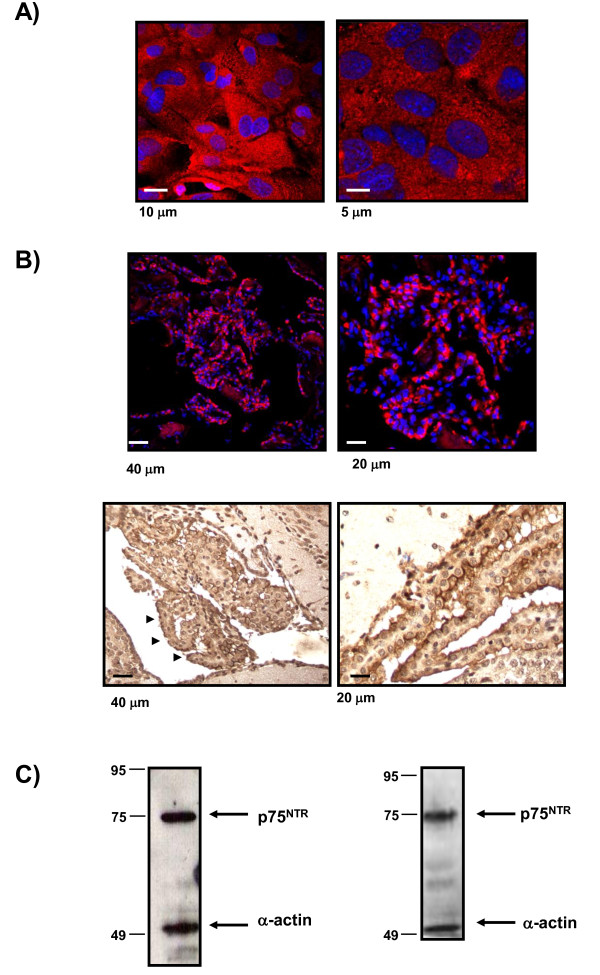
**p75NTR receptor is expressed in the epithelial cells from choroid plexus**. A) Figure 1A shows p75NTR staining of epithelial cells from choroid plexus in *vitro *with p75NTR-ECD antibody (9651) donated by M. Chao and B Carter. The expression is mainly in the cytoplasm of the cells an immunofluorescence (red) and the nucleus is staining with DAPI. B) Figure 1B shows p75NTR staining of human choroid plexus with p75NTR-ECD antibody (9651) (upper panel). The bottom panel shows p75NTR staining developed with DAB and shows the p75NTR expression in the cytoplasm and in the apical membrane of mouse choroid plexus corresponding with CSF side of epithelial cells. C) Western blot analysis of homogenates from primary culture cells from epithelial cells of choroid plexus (left) and mouse choroid plexus tissue (right). Representative blots are shown

Figure 1 shows p75NTR immunofluorescence, in three different species, with a well-established antibody against p75NTR receptor, donated by M. Chao (9651). p75NTR receptor signal was detected in a monolayer of epithelial cells from a choroid plexus primary culture (Figure 1A). p75NTR immunofluorescence staining was also shown in human samples from biopsied choroid plexus (Figure 1B, upper), and in samples of mouse choroid plexus, using 3,3'-Diaminobenzidine (Figure 1B, bottom). The p75NTR expression was specifically detected in the cytoplasm of choroid plexus epithelial cells and mainly located in CSF side of the blood-CSF barrier (Figure 1B). In order to test p75NTR expression by Western blot analysis we lysed different samples of choroid plexus. p75NTR expression was detected in primary cultures of choroid plexus and in tissue of mouse choroid plexus (Figure 1C).

### 2. Proteolytic processing of p75NTR by amyloid-β (Aβ) in choroid plexus

Aβ1-40 aggregates of undetermined structure have been reported to be death-inducing ligands of p75NTR [26]. Although Aβ is also known to bind many other targets, such as megalin, the most important multicargo receptor in the choroid plexus [27]. It has been known for many years that p75NTR undergoes ectodomain shedding. Ectodomain cleavage of full length p75NTR releases the extracellular domain from the membrane bound C-terminal fragment (CTF) ≈24 KDa. The CTF is subsequently cleaved by γ-secretase to give rise to the soluble p75NTR intracellular domain (ICD) ≈19 KDa [28].

**Figure 2:**
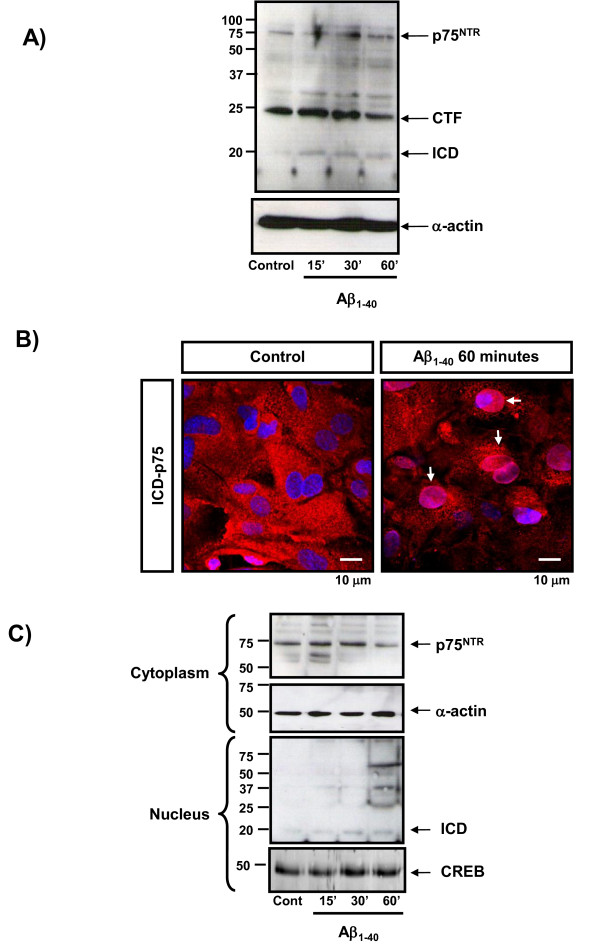
**Proteolytic processing by Amyloid-β (Aβ) in choroid plexus cells**. Western blot analysis of homogenates from primary culture cells from choroid plexus treated with Aβ1-40 2.5 μg/mL during 15, 30 and 60 minutes. The samples were prior applied with proteosome inhibitor during 1 hour. The membranes were developed with an antibody directed against the cytoplasmic domain of p75NTR (ICD) donated by B. Carter and M. Chao (9992). The blot revealed fragments at 24 KDa consistent with the p75NTR-CTF, and a fragment at 19 KDa consistent with p75NTR-ICD, also was observed weakly. The treatment with Aβ1-40 induced a release of ICD fragment at 15 and 30 minutes. α-actin was used as a loading control. B) Similar analysis as in A) but in immunofluorescence treated with Aβ1-40 2.5 μg/mL, 60 minutes. C) Similar analysis as in A) but we separated cytoplasm and nuclear fractions. We observed that p75NTR-ICD was accumulated in the nucleus of the cells after Aβ treatment. α-actin was used as a loading control of cytoplasm fraction and CREB was used as a loading control of nuclear fraction. Representative blots are shown

The cleavage of p75NTR has been followed essentially in transfected cells [29] and in primary Schwann cells [28]. Previous observations with cell lines have suggested that amyloid could regulate the cleavage of p75NTR. To examine whether p75NTR is cleaved endogenously in epithelial cells from choroid plexus, we performed tests in primary culture cells from the choroid plexus epithelium previously applied with proteasome inhibitor (Calbiochem) and then treated with Aβ1-40 (2.5 μg/mL) during 15, 30 and 60 minutes. Western blotting with an antibody directed against the cytoplasmic domain of p75NTR (ICD), donated by B. Carter and M. Chao (9992), revealed an immature underglycosylated form of 45 KDa and fragments at 24KDa consistent with the p75NTR-CTF. A band at 19KDa consistent with p75NTR-ICD fragment was also observed weakly. The treatment with Aβ1-40 induced a release of ICD fragment at 15 and 30 minutes (Figure 2A). The cellular distribution of p75NTR-ICD was determined in these cells by immunofluorescence and confocal microscopy. To avoid instability of the ICD fragment, primary culture of choroid plexus epithelial cells were treated with proteasome inhibitor (Calbiochem) 1.5 hours before treated with Aβ1-40 during 60 minutes. In control cells we observed the intensity of p75NTR-ICD labelling in the most part of the cytoplasm and in some of the nuclei (Figure 2B, left image). In the presence of Aβ1-40, a considerable accumulation of p75NTR-ICD occurred in the majority of nuclei shifted from cytoplasm to nuclei (Figure 2B, right image).

To confirm that the p75NTR-ICD does indeed accumulate in the nucleus in response to Aβ1-40, nuclear and cytoplasmic extracts were subjected to Western blotting with a p75NTR-ICD specific antibody (9992). A 19 KDa band, characterized as p75NTR-ICD fragment [28], was enriched in nuclear extracts from primary culture cells from choroid plexus exposed to Aβ1-40 for 15, 30 and 60 minutes (Figure 2C).

### 3. p75NTR receptor involved in Aβ transport in the choroid plexus

**Figure 3:**
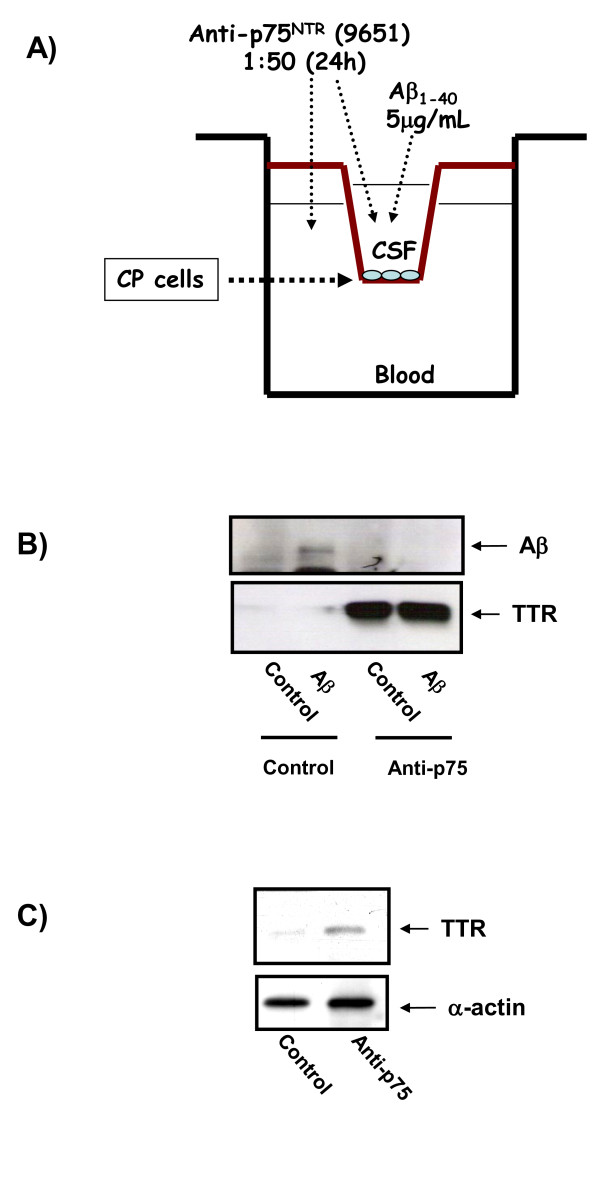
**p75NTR is involved in Aβ transport through choroid plexus**. A double-chamber choroid plexus epithelial cell-culture system mimicking the blood-CSF interface was used for *in vitro *studies A) Figure 3A shows a scheme of one of these chambers. Choroid plexus epithelial cells were grown in the floor of the upper compartment on the top of a porous membrane. Aβ1-40 5 μg/μL was added to the upper chamber. For inhibition of p75NTR biological activity experiments, prior Aβ1-40 were added in the upper chamber, where we had inhibited the p75NTR activity incubating 24 hours with high concentrations (1:50) of p75NTR antibody directed against extracellular domain (9651). B) In control cells Aβ is normally translocated to the lower chamber, however the cells with p75NTR biological activity inhibiting Aβ translocation was interrupted. TTR is a protein specifically synthesized in the brain by the choroid plexus and associated with Aβ transport via megalin in the choroid plexus. Interestingly, we evidenced that the inhibition of p75NTR biological activity induced a strong secretion of TTR to the upper chamber. C) Inhibition of p75NTR biological activity increased TTR expression in the choroid plexus cells. Representative blots are shown.

In order to investigate the influence of p75NTR receptor on choroid plexus function, we used two different methods. Firstly, with *in vitro *double-chamber well used to mimic the blood (lower chamber)-CSF (upper chamber) interface (Figure 3A). Choroid plexus epithelial cells were grown in the floor of the upper compartment on the top of a porous membrane. In other systems the incubation with antibodies against extracellular domains of membrane receptors are able to inhibit the activity of some ligands, due to antibody and ligand compete for the same localization in the receptor. With previous experiments we checked that incubations with high concentrations of p75NTR antibody block the activity of p75NTR in our cell system. Based on this results, prior Aβ1-40 addition in the upper chamber, we had inhibited the p75NTR activity by incubation during 24 hours the upper chamber with high concentrations (1:50) of p75NTR antibody directed against extracellular domain (9651). Indeed, as determined by Western blotting analysis in control situation, Aβ is transported from inner chamber (CSF) to outer chamber (blood) (Figure 3B). Aβ translocation is enhanced through out megalin, as shown by immunoprecipitation analysis (Figure 3B), and was already suggested by Zlokovic's group [27]. When p75NTR biological activity was inhibited in this *in vitro *model with p75NTR antibodies, Aβ CSF-to-blood transport is interrupted (Figure 3B). We then analyzed expression and release of TTR, a protein specifically synthesized in the brain by the choroid plexus [30], and associated with Aβ transport via megalin in the choroid plexus [31]. We observed, in our *in vitro *blood-CSF-barrier model, that the inhibition of p75NTR biological activity induced TTR expression in the choroid plexus (Figure 3C) and strong TTR release in the culture medium of the upper chamber (Figure 3B).

### 4. The p75NTR receptor is implicated in cell death induced by Aβ

**Figure 4:**
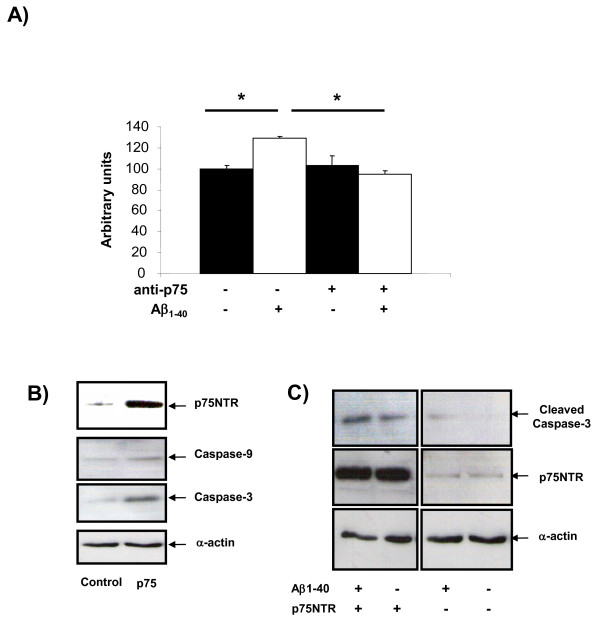
**p75NTR is involved in the cell death induced by Aβ**. A) Cell death quantification with ELISA PLUS Kit. In control cells Aβ induced a 29% of cell death, when we blocked the p75NTR biological activity with prior p75NTR antibody incubation (1:50) during 24 hours, the Aβ did not produce any cell death in choroid plexus. B) Increased levels of caspase-3 and caspase-9 after p75NTR transfection in primary cell cultures. C) Increased of cleaved-caspase-3 in primary culture cells transfected with p75NTR. The choroid plexus culture cells that over-express p75NTR were more sensitive to damage induced by Aβ increasing the cleaved-caspase-3 band in the blot.

In control situation we corroborated that Aβ induced an increase of death cell of 29% upon control cells. When we blocked the biological activity of p75NTR, Aβ-induced cell death was inhibited (Figure 4A). After that, we transfected with p75NTR plasmid primary culture cells of choroid plexus epithelial cells and we corroborated that over-expression of p75NTR receptor induced an increase of caspase-3 and caspase-9 expression (Figure 4B). When we added Aβ we observed the increase of cleaved-caspase-3 in both situations (control and p75NTR over-expression). However, when p75NTR was over-expressed, the cleaved-caspase-3 was strongest compared with control (Figure 4C).

## Discussion

The p75NTR receptor is a transmembrane protein that binds all neurotrophins and has multiple functions in the nervous system, where it is widely expressed during developmental stages of life in neurons and also in a variety of glial populations. Expression of p75NTR receptor can increase in pathological states related to neural cell death. In choroid plexus only transcripts coding for the TrkB molecule have been described [23]. Our data clearly demonstrate that p75NTR receptor is also expressed in the choroid plexus and is mainly located towards the apical membrane in the epithelial cells. In Madine-Darby canine kidney (MDCK) cells transfected with the plasmid p75NTR receptor, the expression is restricted to the apical domain [32]. Several studies demonstrated that p75NTR participates in more diverse biological events including neuronal cell death, migration and axonal elongation. In the present study we have suggested the role of p75NTR receptor in the epithelial cells from choroid plexus and it seems to be involved in the damage and death of cells induced by Aβ.

It is well described in neurons and different cells lines that p75NTR receptor undergoes sequential proteolytic cleavage by α-secretase and γ-secretase activities, releasing its ICD into cytoplasm, which is in a manner analogous to the cleavage-dependent signalling pathway of Notch and APP [29]. Although neurotrophins did not regulate p75NTR processing, the α-secretase and γ-secretase mediated cleavage of p75NTR is modulated by TrkA and TrkB receptors [33] and the choroid plexus epithelial cells were able to express TrkB receptors [23]. In this context, we have presented evidence that the predicted ICD of p75NTR was detectable by Western blot analysis and immunostaining after treatment with Aβ, and this fragment was translocated to the nucleus. The ICD of p75NTR was unstable in the choroid plexus; we therefore attempted to detect it by inhibiting a proteasomal pathway that had been previously shown to mediate degradation of Notch-ICD or p75-ICD in neurons [34]. We have not evidence about the function of ICD nuclear accumulation in choroid plexus, however, there were many evidences that p75NTR-ICD have nuclear functions [35], such as apoptosis [36], transcriptional and cell cycle regulations [37]. Numerous proteins interact with p75NTR-ICD to activate different pathways (NF-kappaB, Akt or JNK) and some of them have been associated with the translocation to the nucleus and the induction of apoptosis [38]. However we have not evidence that some of these adaptors, such as NRIF, TRAFs, SC1, MAGE, RIP2, are expressed in choroid plexus. It remains to be determined whether adaptor proteins of p75NTR are also expressed in choroid plexus and can interact with p75NTR. The identification of p75NTR interactors and signaling pathways will spark new directions in blood-CSF-barrier research and will provide better understanding of this enigmatic receptor.

The choroid plexus play pivotal roles in basic aspects of neural function including maintaining the extracellular milieu of the brain by actively modulating chemical exchange between the CSF and brain parenchyma. In this context, it is well known the role of megalin, a multifunctional endocytic receptor. Megalin is a multicargo transmembrane protein with a large extracellular domain containing multiple binding sites for its numerous ligands. Megalin is the major receptor for the uptake of Aβ complexed with different proteins, such as TTR, clusterin, ApoE, albumin [39,40]. Based on previous observations, megalin mediated clearance mechanisms of Aβ have been proposed to play a crucial role in the elimination of Aβ from the brain through the blood-CSF-barrier into the periphery [27,41]. To investigate the possible role of p75NTR receptor in an artificial model of blood-CSF-barrier, we corroborated the normal Aβ transport from CSF to blood; however, when we inactivated the biological activity of p75NTR we demonstrated that Aβ transcytosis was completely blocked. These results suggest that p75NTR receptor could be modulating megalin activity in the blood-CSF-barrier, and modifying the Aβ clearance. The precise mechanism as to how the p75NTR modified megalin activity remains elusive.

Our findings have led to the suggestion that the p75NTR signaling in choroid plexus might be determined by the type of co-receptor involved in the complex. Our studies revealed that Aβ could be able to engage a functional interaction between p75NTR and megalin. Further, TrkB expression was detected in choroid plexus [23], and in neurons it was well know to engage functional interaction between p75NTR and TrkB [42]. The mechanism of TrkB and p75NTR in choroid plexus remains elusive and needs more experiments; however we suggest that the possible interaction between p75NTR, TrkB and megalin will provide new directions in the p75NTR signaling and better understanding in the blood-CSF-barrier research.

The mechanism underlying this phenomenon and its relevance to neurodegenerative diseases is unclear. Therefore, it is reliable to speculate that p75NTR receptor could modify TTR expression and release it to CSF. Previous studies confirmed by data from our laboratory suggested that TTR is reduced in CSF samples from subjects with Alzheimer's disease and frontotemporal dementia [43]. Experiments investigating the response of choroid plexus TTR synthesis after inhibition of p75NTR biological activity produced an increase of TTR synthesis; alternatively when we investigated the inhibition of p75NTR biological activity in an *in vitro *model of blood-CSF-barrier, we confirmed a strong release of TTR (upper chamber). It is well established in our laboratory that Aβ induced cell death in choroid plexus epithelial cells [4]. We confirmed the implication of p75NTR receptor in Aβ-induced cell death by blocking the p75NTR biological activity. These experiments were corroborated when we over-expressed p75NTR receptor in primary culture of choroid plexus epithelial cells increasing the expression of caspase-3 and caspase-9, and cleaved of caspase-3. Dietrich's study shows Aβ accumulation in choroid plexus in different stages of Alzheimer's disease [44] and induced oxidative stress and mitochondrial alterations [45]. These results showed the impact of p75NTR receptor in the survival and protection of epithelial cells from choroid plexus and confirmed the inhibition of p75NTR biological activity such as a protective mechanism in neurodegenerative diseases, but further studies using animal models will be required to confirm this hypothesis.

## Conclusions

In summary, our results have been revealed p75NTR expression in epithelial choroid plexus, mainly localized in the apical membrane in contact with CSF side. Indeed, p75NTR plays an important role in cell surface platforms to induce neuronal apoptosis [25]. In our context we suggest that p75NTR is also regulating the survival/apoptosis pathways and the Aβ-induced damage. The mechanism of TrkB and p75NTR in choroid plexus remains elusive and needs more experiments, although we suggest that p75NTR and TrkB could interact with megalin providing new directions in the p75NTR signaling and better understanding in the blood-CSF-barrier research.

## Methods

### Cell Cultures

Primary cultures from choroid plexus epithelial cells from P3-P5 Wistar rats were prepared as described previously [11]. Cells were grown to confluence for 5-7 days and serum starved for 2 hours. Cultures were maintained at 37°C in a humidified atmosphere containing 5% CO2, and cultivated for 7 days prior to experimentation. Human analogue peptides corresponding to Aβ1-40 and scrambled Aβ1-40 (AnaSpec, Inc.) were added. 24 hours after stimulation, cells were either fixed for immunocytochemical analysis or homogenized for immunoblot determination. For ICD p75NTR detection primary cultures cells proteasome inhibitor epoxomycin (1 μM) (Sigma) was applied 1.5 hours prior to Aβ treatment.

A double-chamber choroid plexus epithelial cell-culture system mimicking the blood-CSF interface was used for *in vitro *studies, as described previously [11].

Thereafter, cells were incubated another 24 hours with excess of p75NTR (9651) antibody (1:50) (gifted from Dr. M. Chao and Dr. B. Carter). The antibody was added to the upper and lower chamber and 24 hours later the upper chambers were treated with Aβ1-40 (5 μg/mL). Twenty-four hours later, lower chamber medium was collected and content of Aβ and TTR was determined by immunoblotting (see below). Treatments were done in triplicate wells per experiment.

All animals were handled and cared for in accordance with European Community Council Directive (86/609/EEC). Animals were perfused transcardially with saline buffer and 4% paraformaldehyde in 0.1 M phosphate buffer, pH 7.4, for immunohistochemical analysis.

### Immunoassays

For Western blot analysis cultures cells were washed once with ice-cold PBS and lysed in PIK buffer (150 mM NaCl, 20 mM TrisHCl pH 7.4, 1% NP40 and protease inhibitors: 1 μg/mL aprotinin, 1 μg/mL leupeptin and 1 μg/mL phenylmethylsulfonyl fluoride, PMSF). For cell fractionation cells were lysed with C buffer (10 mM HEPES, 60 mM KCl, 1 mM EDTA, 0.075% Triton X100, 1 mM DTT with protease inhibitors. The pellet nuclei was obtained by centrifugation 325 g, 4 minutes and resuspended in NB buffer (20 mM TrisHCl pH 8, 420 mM NaCl, 15 mM MgCl2, 0.2 mM EDTA, 25% Glicerol and protease inhibitors). Thereafter, the nuclei supernatant were obtained after centrifugation 9000 g, 10 minutes. After running the samples in acrylamide gels, proteins were transferred (immobilon, Bio-Rad) and membranes were incubated with the corresponding primary at 4°C overnight. Afterwards, membranes were washed and incubated with secondary antibodies. Membranes were washed several times with Tween-TBS and developed with ECL plus (Amersham). Western blot membranes were re-blotted with unrelated proteins (α-actin) as an internal standard and normalized for protein load. Densitometric analysis was performed using ImageJ software (NIH Image). A representative blot is shown from a total of at lest three independent experiments.

For immunoprecipitation, cells were lysed with PIK buffer and centrifuged at 11000 g for 15 minutes. Supernatants were incubated with primary antibody overnight. Protein A-agarose (Amersham) was added to the antigen-antibody mixture and incubated with gentle agitation 4 hours. The immunoprecipitate was washed several times with the same PIK buffer, resuspended in SDS loading buffer and analyzed by western blot.

For cell death quantification, after incubation for 24 h with Aβ40 (5 mg/ml), choroid plexus epithelial cells *in vitro *were measured with Cell Death Detection ELISA_PLUS _kit (Roche Diagnostics) as described previously [4].

### Immunofluorescence

Immunocytochemistry was performed with primary cultures from choroid plexus plated on 20 mm coverslips and fixed. Rat and human choroid plexus were also fixed with 4% paraformaldehyde and processed for histochemical analysis. Coverslips and choroid plexus sections were blocked with 5% BSA and incubated overnight at 4°C with the respective antibody in PB containing 0.5% BSA and 0.1 Triton X100. After several washes in PB, sections were incubated with Alexa-coupled secondary antibody in the same PB buffer. Omission of primary antibody was used as control. Confocal analysis was performed in a Leica confocal microscope. Human brain tissue was obtained from the Neuropathology Institute, anatomy pathology service, IDIBELL. Hospital "Universitario de Bellvitge" (Barcelona, Spain).

### Antibodies

The following antibodies were used: rabbit polyclonal anti-p75NTR against extracellular domain (9651) and intracellular region (9992) gifted from Dr. M. Chao and Dr. B. Carter. Mouse monoclonal anti-α-actin (Sigma), mouse monoclonal anti-β-amyloid (MBL), rabbit polyclonal anti-transtyrretin (Santa Cruz), mouse monoclonal anti-caspase 3 and 9 (Cell Signalling), goat polyclonal anti megalin (Santa Cruz). All alexa fluor antibodies were purchased from Invitrogen.

## Competing interests

The authors declare that they have no competing interests.

## Authors' contributions

Conceived and designed the experiments: CS and EC. Performed the experiments: CS. Analyzed the data: CS and EC. Wrote the paper: CS. CS and EC read and approved the final manuscript.
